# The use of restrictive practice in the care of people living with dementia during a hospital admission in the UK: Results of an ethnographic study

**DOI:** 10.1002/alz70860_099731

**Published:** 2025-12-23

**Authors:** Andy Northcott, Katie Featherstone, Shadreck Mwale

**Affiliations:** ^1^ Geller Institute of Ageing and Memory, University of West London, London, London, United Kingdom; ^2^ Geller Institute of Ageing and Memory, the University of West London, London, London, United Kingdom

## Abstract

**Background:**

People living with dementia are at significant risk of experiencing restrictive practice or restraint during an unscheduled acute hospital admission, however it has remained an unexplored feature of routine care. In the UK hospital data recording the use of restrictive practice is poor, highlighting that these practices are poorly recognised and under reported, to the detriment of high‐quality care.

**Method:**

This paper draws on 225 days of ethnographic (observational) research exploring the use of restrictive practice in the care of people living with dementia across a hospital admission. Our observational data was collected over 18 months from nine wards in three regions of England, covering a range of geographies and socio‐economic demographics. In each region an assessment unit (30 days), an older persons care ward (30 days) and a specialist mental health ward (15 days) were observed, alongside in‐situ interviews with patients and staff delivering care.

**Result:**

This study identified restrictive practice as an invisible but everyday feature of care experienced by all people living with dementia within hospital wards. While quantifiable and visible restraint (physical restraint, pharmaceutical sedation, legal frameworks) was observed, our findings highlight that hidden, unrecognised, and unrecorded, restraint was experienced by all people living with dementia admitted to hospital. This encompassed a wider range of routine practices, staff attitudes, and ward cultures of control and containment, during care.

**Conclusion:**

People living with dementia are expected to remain in bed or at the bedside across an acute hospital admission, with opportunities to leave the bedside further reduced when restrictive practices were applied, with significant impact on the person, causing high levels of distress, and poor outcomes. Changes to cultures of care and alternative approaches to care are needed to improve the experiences and outcomes of a hospital admission for this significant and vulnerable population within our hospitals.

